# Castration for Crime as Preventive and Curative Treatment

**Published:** 1896-03

**Authors:** J. W. Frazier

**Affiliations:** Belton, Texas


For Texas Medical Journal.

### CASTRATION FOR CRIME AS PREVENTIVE AND CURATIVE TREATMENT.

BY J. W. FRAZIER, M. D., BELTON, TEXAS.

Read before Central Texas Medical Society at Waco, January 15, 1896.

VICE or crime from a psychological, sociological, medico-legal and moral standpoint may be appropriately considered a true disease, i. e., a variation from the normal healthy and usual cause of action.

Like many other diseases with which our therapeutics claim familiarity, notably, chorea, hysteria and epilepsy, we may not be able to locate the lesion or intelligently determine its pathology; yet the scientific discussion of its etiology and the suggestion of appropriate remedies comes within the province of our profession. Indeed Benedict says the brains of criminals deviate essentially from the normal type, not so much in form (for there is probably no typical abnormal shape of brain peculiar to criminals), but in structnre. This is perhaps the one necessary feature present in the brains of those belonging to the criminal classes.

That it may be changed by long continued scientific and persistent efforts at reformation is doubtless true, but when and where are such efforts to be made? They never have been, and probably never will be undertaken, and the criminal as usually discharged from prison or reformatory is certainly no better, and often far worse than when he entered.

Obedience to the laws and customs of his country and a recognition of the rights of his fellows is an inherent, innate principle in the normal healthy mind. Crime presupposes either an inability to differentiate between a right and a wrong course of action, or knowing the difference, a lack of neurotic inhibitory resisting force.

In considering the etiology of crime in general, we must consider the emotional instinct, denominate love. Love of self and love of the opposite sex furnish probably the greatest of all motives for human action. This passion or instinct properly directed and kept within due bounds, enables man to attain the highest aims and purposes. But misdirected or inordinately developed, it is directly or indirectly one of the most important etiological factors leading to criminal action.

An attribute of crime especially important when we come to discuss a remedy, is *heredity*. To the student of sociology, this is an important and thoroughly established fact, and the criminal is a menace, not only to the society and generation in which he lives, but can transmit his moral defects to an endless posterity; and this without let or hindrance in the way of legal restraint.

But hereditary or acquired, the criminal is and always has been a menace to the social fabric, and its prevention and punishment or treatment has engaged the best minds in all ages. Remedies suggested have been embodied in criminal statutes providing punishment supposed to be commensurate with the character of the crime.

The motive prompting such legal enactments in civilized countries is, or should be, not so much a spirit of revenge as a means of preventing a repetition of the criminal act.

Now a rigid, strict and impartial administration of the criminal laws already in force, would undoubtedly remedy in some degree the dreadfully increased criminal tendency of the age. Swift and certain justice following the commission of a crime would be an object lesson our criminal classes would be quick to learn and heed.

The inefficiency of the present statutory provisions to prevent crime is patent to the most casual observer of current events, even presupposing their rigid application and enforcement.

As a more radical, rational scientific remedy, preventive and curative of crime, the present paper is intended to suggest and earnestly advocate castration.

As a penalty for crime, this remedy has never been engrafted in the criminal statutes of any civilized country within my knowledge.

Yet its suggestion is no innovation. Dr. Agnew suggested, in 1887, that emasculation, as a penalty for the crime of rape, would be followed, if inflected legally, by satisfactory results.

Enlarging on this suggestion, in a scholarly and classical paper read before the Cincinnati Academy of Medicine, February 27, 1887, Dr. Orpheus Everts formulated this proposition: "Surgical asexualization of all criminals convicted of offenses that, circumstantially considered, indicate constitutional depravities that are recognized as transmissible by heredity, is not only practical, but expedient, for the protection of society against the ever impending danger of invasion by the 'savages of civilization,' known as the vicious, criminal or defective classes, and would, properly enforced by law, eventuate in an effectual elimination of crime and reformation of criminals."

Dr. Wm. A. Hammond read a very able paper before the N. Y. Society of Medical Jurisprudence March 14, 1892, entitled, "A New Substitute for Capital Punishment and Means of Preventing the Propagation of Criminals."

While in 1893 appeared the very excellent paper of our own Dr. F. E. Daniel, entitled, "Castration of Sexual Perverts," read before the American Medico-Legal Society of New York, and the International Medico-Legal Congress, sitting at Chicago in 1893. In the same year appeared the noted correspondence between Dr. Hunter McGuire, of Richmond, Virginia, and Dr. Lydston, of Chicago, on "Sexual Crimes among the Southern Negroes, Scientifically Considered."

Dr. F. L. Sim, of Memphis, in a paper entitled, "Asexualization for the Prevention of Crime and arrest of the Propagation of Criminals," read before the Tennessee State Medical Association, April, 1894, and Dr. B. A. Arbogast, of Colorado, in a paper, "Castration the Remedy for Crime," read before the Colorado State Medical Association, August, 1895, are earnest and zealous advocates of this new remedial agent for crime.

That castration has a very grave alterative effect on the brain tissue of the individual castrate, is a well recognized scientific act. The whole nature of the man undergoes a very radical change, so that the "lean and hungry Cassius" with a mind "fit for treason, strategy and spoils," becomes a fat, lazy, inoffensive nonenity, from whom society shrinks, but feels no apprehension.

Among the domestic animals, the radical alteration brought about by castration is a matter of common observation. The stockman who owns a vicious bull or stallion, castrates him at once, and thus converts him into a docile, tractable and useful animal. The history of the eunuch of former times shows that he not only lost his licentiousness, but all feeling of manhood and equality as well, and grew fat from an easy conscience and a tranquil nervous system.

Dr. Hammond says, in the paper before referred to: "Were castration the punishment for murder, it would be perfectly safe to allow the criminal upon whom the operation had been performed, to go at large, with entire confidence that he would be thereafter indisposed to homicidal acts, or indeed violence of even less degree."

Indeed, castration creates such a notable change in the man upon whom it is performed, that this great alteration can only be accounted for on the hypothesis that it has produced a profound modification of the brain structure, and this to such an extent as to remove the subject of the operation from the criminal class, and to place him in such a condition that great crimes and misdemeanors are entirely beyond his desire and capacity. As a reforming agent, it stands unequaled by all the effects that it is possible for us to make through other, less radical agencies. The punishment is, in my opinion, more deterrent than that of death, more advantageous to the State than either death or imprisonment, and more powerful as a reformatory agent than all the teachings and examples that sociology can offer.

For all so-called sexual crimes the remedy is peculiarly appropriate, rational, scientific and just. The highest, and unfortunately, most common of this class of crimes, is that of rape, and this, notwithstanding the fact that our laws at present provide the severest penalty known to man for this crime; and the ungovernable passions of an outraged community add often to the horrors of the statutory death penalty. This is a remedy effectual as to the individual crime, but statistics and the daily record of current events show that rape is horribly on the increase.

So, likewise, of other lesser criminal acts of sexual perverts.

The proper and scientific remedy for a perverted sexual appetite is the radical removal of the means of gratifying the criminal desire; render the criminal sterile by castration, thorough and complete. The remedy should rationally be extended to a vast category of other crimes, and in some cases added to the punishment already provided for. The vicious and depraved of the confirmed and convicted criminal class should be deprived of the capacity to procreate in kind, as a protection to the society of posterity. Then, too, the best interests of the individual criminal are conserved, and an opportunity given to reform; deprived of his mania to commit offense to his fellows, he is made a living, ever present, object lesson to others with like tendencies, and thus worth more as a moral inhibitory force than a hundred capital punishments or penitentiary convictions, usually the talk of a narrow circle for a few days, then relegated to oblivion. The contemplation of a sure castration on conviction would have a much greater deterrent effect on the mind of one who contemplated doing a willful murder, rape, arson, or robbery, or other grave offense, than any other punishment that could be devised. The mental organization of this class approaching more the brutal instincts, the gratification of sexual desires, the dread of the loss of this function would, in my opinion, be a far greater deterrent factor than the loss of life itself.

Conceding the efficiency of the remedy, what are the obstacles to its acceptance?

First and foremost, public opinion. From the earliest times the public, it seems, has placed the generative organs of man upon a pedestal and held them sacred. You may take the life or liberty of the criminal, but you must not so much as touch his testicles.

Now, in the light of science and of reason, this is absurd and can be overcome by an intelligent agitation of the subject, so that the thinking classes of the community can be brought to understand its bearings, and the special advantages of castration over death or imprisonment for life.

I know that it is claimed by some legal authorities that the infliction of such a penalty would be unconstitutional.

On this point Col. A. C. Holt, a distinguished Southern legal authority, says, there is no clause in the Federal or State Constitution which forbids punishment of that character; while the Hon. Clark Bell, in discussing Dr. Daniel's paper before the International Medico-Legal Congress, says: "There can be no doubt whatever of the legal right of the State to pass laws to make this act (castration or asexualization) a punishment for crime, and to insert this penalty in the methods of punishmen in the category of punishments for crime. On that there can not be the slightest difference among lawyers and jurists."

Now, gentlemen, in conclusion; should the importance and magnitude of the subject impress itself upon the minds of the members of this Association as it has upon mine, I trust they will, as an organization, adopt such measures as in their wisdom they may see fit to bring the matter before the public and the State legislature for its consideration and adoption.

---

